# Baseline characteristics and risk factors for ulcer, amputation and severe neuropathy in diabetic foot at risk: the BRAZUPA study

**DOI:** 10.1186/s13098-016-0126-8

**Published:** 2016-03-17

**Authors:** Maria Candida R. Parisi, Arnaldo Moura Neto, Fabio H. Menezes, Marilia Brito Gomes, Rodrigo Martins Teixeira, José Egídio Paulo de Oliveira, Joana Rodrigues Dantas Pereira, Reine Marie Chaves Fonseca, Lorena Barreto Arruda Guedes, Adriana Costa e Forti, Ana Mayra Andrade de Oliveira, Marta Barreto de Medeiros Nóbrega, Víctor Nóbrega Quintas Colares, Helena Schmid, Otto Henrique Nienov, Marcia Nery, Túlio Diniz Fernandes, Hermelinda C. Pedrosa, Cristina da S. Schreiber de Oliveira, Marcelo Ronsoni, Karla Freire Rezende, Maria Teresa Verrone Quilici, Alexandre Eduardo Franzin Vieira, Geisa Maria Campos de Macedo, Eliana Gabas Stuchi-Perez, Kandir Genésio Innocenti Dinhane, Ana Emilia Pace, Maria Cristina Foss de Freitas, Maria Regina Calsolari, Mario José Abdalla Saad

**Affiliations:** Faculty of Medical Sciences, State University of Campinas, Campinas, SP 13083-887 Brazil; Unity of Diabetes, State University of Rio de Janeiro, Rio de Janeiro, Brazil; Department of Nutrology, Federal University of Rio de Janeiro, Rio de Janeiro, Brazil; Centre for Diabetes and Endocrinology in the State of Bahia (CEDEBA), Salvador, Brazil; Centro Integrado de diabetes e Hipertensão do Ceará, Fortaleza, Brazil; State University of Feira de Santana, Feira de Santana, Brazil; Federal University of Campina Grande, Campina Grande, Brazil; Hospital das Clínicas de Porto Alegre, Federal University of Rio Grande do Sul, Porto Alegre, RS Brazil; Santa Casa de Porto Alegre, Porto Alegre, RS Brazil; Hospital das Clínicas da Faculdade de Medicina, Universidade de São Paulo, São Paulo, Brazil; Hospital Regional de Taguatinga, Brasília, Brazil; Faculdade de Medicina, Federal University of Santa Catarina, Florianopolis, Brazil; Federal University of Sergipe, São Cristóvão, Brazil; Pontifícia Universidade Católica de Sorocaba, Sorocaba, Brazil; Division of Endocrinology, Hospital Agamenon Magalhães, Recife, Brazil; Faculdade de Medicina de Catanduva, Catanduva, Brazil; Faculdade de Medicina de Botucatu, UNESP, Botucatu, Brazil; Universidade de São Paulo, Faculdade de Medicina, Ribeirão Preto, Brazil; Santa Casa de Belo Horizonte, Belo Horizonte, Brazil

**Keywords:** Diabetes, Risk factors for ulcer, Amputation, Severe neuropathy, Brazil

## Abstract

**Background:**

Studies on diabetic foot and its complications involving a significant and representative sample of patients in South American countries are scarce. The main objective of this study was to acquire clinical and epidemiological data on a large cohort of diabetic patients from 19 centers from Brazil and focus on factors that could be associated with the risk of ulcer and amputation.

**Methods:**

This study presents cross sectional, baseline results of the BRAZUPA Study. A total of 1455 patients were included. Parameters recorded included age, gender, ethnicity, diabetes and comorbidity-related records, previous ulcer or amputation, clinical symptomatic score, foot classification and microvascular complications.

**Results:**

Patients with ulcer had longer disease duration (17.2 ± 9.9 vs. 13.2 ± 9.4 years; p < 0.001), and poorer glycemic control (HbA1c 9.23 ± 2.03 vs. 8.35 ± 1.99; p < 0.001). Independent risk factors for ulcer were male gender (OR 1.71; 95 % CI 1.2–3.7), smoking (OR 1.78; 95 % CI 1.09–2.89), neuroischemic foot (OR 20.34; 95 % CI 9.31–44.38), region of origin (higher risk for those from developed regions, OR 2.39; 95 % CI 1.47–3.87), presence of retinopathy (OR 1.68; 95 % CI 1.08–2.62) and absence of vibratory sensation (OR 7.95; 95 % CI 4.65–13.59). Risk factors for amputation were male gender (OR 2.12; 95 % CI 1.2–3.73), type 2 diabetes (OR 3.33; 95 % CI 1.01–11.1), foot at risk classification (higher risk for ischemic foot, OR 19.63; 95 % CI 3.43–112.5), hypertension (lower risk, OR 0.3; 95 % CI 0.14–0.63), region of origin (South/Southeast, OR 2.2; 95 % CI 1.1–4.42), previous history of ulcer (OR 9.66; 95 % CI 4.67–19.98) and altered vibratory sensation (OR 3.46; 95 % CI 1.64–7.33). There was no association between either outcome and ethnicity.

**Conclusions:**

Ulcer and amputation rates were high. Age at presentation was low and patients with ulcer presented a higher prevalence of neuropathy compared to ischemic foot at risk. Ischemic disease was more associated with amputations. Ethnical differences were not of great importance in a miscegenated population.

## Background

The rising prevalence of diabetes around the world has dramatically increased the number of people bearing complications of this potentially incapacitating disease [1]. Peripheral neuropathy and diabetic foot are among the most feared. They frequently terminate in ulcer, infection and amputation, significantly reducing quality of life [2]. The life expectancy of a person with lower limb amputation is comparable to that of other serious diseases, such as aggressive types of cancer or advanced congestive heart failure [3, 4].

Additionally, it is estimated that one person undergoes amputation every 20 s around the globe as a complication of diabetes and that up to 85 % of these procedures could be prevented with adequate screening [5].

The incidence of diabetes is rising more rapidly in developing regions such as Latin America and Southeast Asia than in Western Europe or North America [1, 6], putting a serious economic burden on the healthcare budgets of these nations. Given the disparities in health care access and the social, economic and ethnic differences between these regions, it is crucial to gain knowledge of the clinical characteristics and epidemiology of foot at risk in different areas of the world.

Additionally, several studies have highlighted the dissimilarities in diabetes incidence, management, prevalence and metabolic control, as well as risk and clinical evolution of foot complications [7, 8]. Studies on foot ulcers involving a significant and representative sample of patients such as the EURODIALE cohort [9] are scarce, especially in developing regions. In Brazil, in addition to the lack of descriptive data on foot at risk and ulcer prevalence, there are great dissimilarities between different regions regarding access to health care and socioeconomical structure that could lead to different presentations of foot at risk and diabetic foot complications.

Therefore, our aims were to acquire data on the clinical and epidemiological characteristics of a large cohort of patients from several centers in our country, all specializing in the evaluation and care of patients with diabetes and focus on factors that could be associated with the risk of ulcer and amputation.

## Methods

**Fig. 1:**
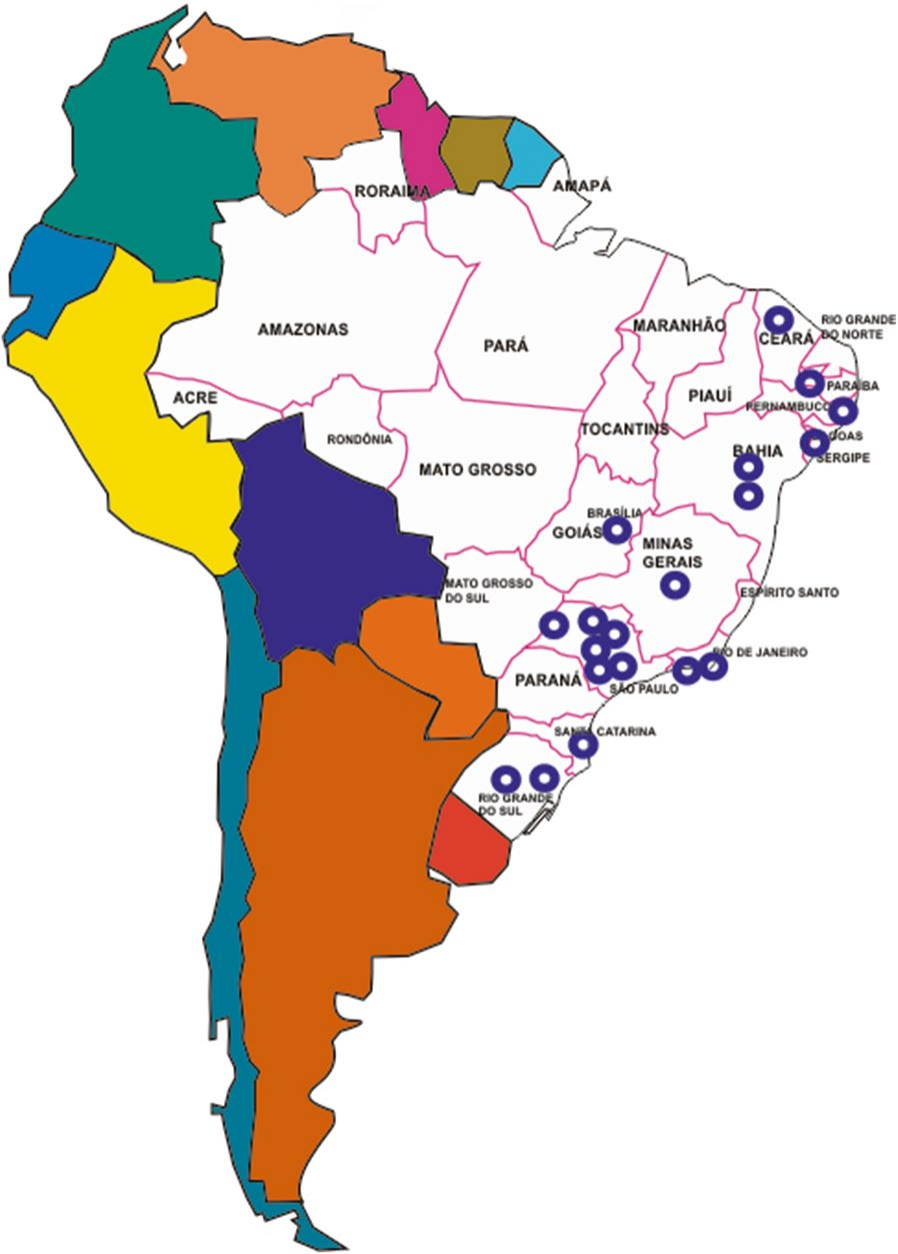
Geographical distribution of the centers

This study presents cross sectional, baseline results of the Brazilian Cooperative Study on Ulcer, Severe Peripheral Neuropathy and Amputation (BRAZUPA), which is funded by the National Institute of Science and Technology—Obesity and Diabetes Division, in order to gather data on the current situation of foot at risk throughout the nation. One thousand fifty-five consecutive patients referred for evaluation of foot at risk to 19 different specialized diabetic centers in the country were included in the study. Among these, six included diabetic foot care units and the others started to structure theirs after the study beginning. Patients were referred from primary care facilities according to Brazilian National Health System (SUS) standard proceedings. All data were collected between June 2012 and July 2014 and each center submitted data collected during the first year after study entry. The four most populous of the country’s five administrative regions were included, and the data can be considered representative for the whole country (around 200 million people at the time the study was initiated)—Fig. 1.

Trained personnel collected data during patients’ routine evaluations and from the medical records of each institution. Data were collected on a standardized electronic sheet developed during a 6-month long pilot study involving 14 centers, simplified to allow input without compromising the routine care of patients. Dedicated personnel were trained in each center during the pilot study. The data collected was submitted to the coordinating center at the end of the first year (University of Campinas, Campinas, Sao Paulo, Brazil), where it was analyzed as a whole after all centers had submitted their information. Patient characteristics recorded included age, gender, ethnicity (white Latin, African descent, mixed ethnicity and Asian), diabetes and comorbidity related records, previous ulcer or amputation, symptom characteristics and clinical symptomatic score.

Foot at risk was classified as without risk, neuropathic, ischemic or neuroischemic. Neuropathy was defined as a positive result in clinical or symptomatic scores and/or an abnormal sensitivity test using a 10 g Semmes–Weinstein monofilament. Vibratory sensation was classified as present, diminished or absent according to the results of the 128 Hz tuning fork examination [5]. Ischemia was defined as diminished or absent pedal pulses or decreased ankle-brachial pressure index (ABI < 0.9) [5, 10]. Amputations were classified as minor (below the ankle) or major (ankle or higher). Ulcers were evaluated for their prevalence and etiological origin: ischemic, neuropathic or neuro-ischemic.

The presence of other microvascular complications was also evaluated. Retinopathy was assessed by standard fundoscopy by a trained ophthalmologist at each center and nephropathy by the presence of microalbuminuria and/or abnormal creatinine clearance (calculated using the Cockroft-Gault formula). Visual impairment was defined as abnormal Snellen chart test result despite the use of corrective lenses. Renal impairment was defined as creatinine clearance <60 ml/min/1.73 m^2^.

The study was approved by each institution’s ethics in research committee as well as by the coordinating center’s Ethics Committee.

### Statistical analysis

Categorical data is shown as percentages; continuous variables as means (standard deviation—SD).

Comparison between groups of categorical data was made using the Chi Square test. Differences in continuous variables between two groups were evaluated by Student’s t test.

Independent risk factors for ulcer and amputation were assessed by multiple logistic regression analysis. The model for ulcer included age, gender, type of diabetes, disease duration, ethnicity, BMI, smoking status, foot at risk classification (foot without risk as reference category), vibratory sensation, visual impairment, hypertension, renal impairment and macro region of origin. Adjustments were done by the forward conditional method. The model for amputation included all of the abovementioned variables with the addition of history of previous ulcer episode.

Statistical analysis was performed with SPSS version 20.0 (IBM Inc.). Significance was defined as a p value <0.05.

## Results and discussion

### Patient characteristics

Baseline patient characteristics are summarized in Table 1. The mean age and disease duration of participants was 57.67 (14.23) years and 14.21 (9.77) years, respectively. The majority of patients were female (58.6 %), White (53.8 %) and had type 2 diabetes (90.7 %). Nearly a quarter (25.3 %) of patients had a previous history of ulcer and 13.7 % of amputation (17.3 % of these were major amputations). One in every twenty patients (5.3 %) had undergone more than one amputation procedure. One-third of patients had neuropathic foot (33.7 %) and only 8.5 % had ischemic disease alone. Nearly one-fifth (18.6 %) of all evaluated patients presented a current, active foot ulcer at evaluation.

**Table 1:** Clinical and epidemiological characteristics of study participants

Variable	Mean (SD)/%
Age	57.7 (14.2)
Sex (female/male)	58.6/41.4
BMI (kg/m^2^)	29.3 (6.2)
Ethnicity	
White	53.8
Black	18.4
Mixed	20.8
Asian	7.0
Type of diabetes (1/2)	9.3/90.7
Diabetes duration (years)	14.2 (9.8)
HbA1c (%)	8.56 (2.03)
Smoking	24.2
Hypertension	81.5
Cardiopathy	28.5
Dyslipidemia	72.9
Nephropathy	
Stage 1	52.1
Stage 2	25.4
Stage 3	15.8
Stage 4	1.9
Stage 5	4
Retinopathy	46.2
Visual impairment	50.3
Previous ulcer	25.3
Active ulcer	18.6
Amputation	13.7
Minor	82.7
Major	17.3
Foot at risk classification	
Foot without risk	37.8
Neuropathic	33.7
Ischemic	8.5
Neuroischemic	20
Vibratory sensation	
Normal	55.7
Diminished	29.9
Absent	14.5
Score of symptoms	
Normal	20.4
Mild	29.6
Moderate	28.1
Severe	22
Treatment of neuropathy (yes)	14.1
Region	
South	18.9
Southeast	38.9
Midwest	3.4
Northeast	38.8

### Differences between patients with and without amputation, ulcer and mild versus moderate/severe neuropathy

#### Ulcer

We observed a significantly larger proportion of men with a previous history of ulcer (35.7 vs. 18 %; p < 0.001). These patients also had longer disease duration (17.2 ± 9.9 vs. 13.2 ± 9.4 years; p < 0.001), and poorer glycemic control (HbA1c 9.23 ± 2.03 vs. 8.35 ± 1.99; p < 0.001). Those of White or Mixed ethnicities had a higher proportion of ulcer than Black and Asian ethnicities (29 and 24.6 vs. 19.7 and 16.5 %, respectively; p = 0.008). There was a higher prevalence of previous ulcer in those with retinopathy (38.3 vs. 14.1 %; p < 0.001), visual (30.8 vs. 18 %; p < 0.001) and renal impairment (34.8 vs. 22.7 %; p < 0.001). Ulcer was less common in patients with ischemic foot than neuropathic or neuro-ischemic classifications (14.2 vs. 39.3 and 45.1 %, respectively; p < 0.001). Individuals from the economically developed regions (South/Southeast) had a higher prevalence of previous ulcer episode than those coming from economically emerging ones (Midwest/Northeast) (28.8 vs. 20.7 %, respectively; p = 0.001).

Table 2 shows the prevalence of different clinical characteristics in patients with and without ulcers.

**Table 2:** Prevalence (%) and means of clinical characteristics in patients with and without previous ulcer

Variable	Previous ulceration		p
	Yes	No	
Age (years)	58.4	57.5	0.26
Sex (female/male)	41.7/58.3	64.4/35.6	<0.001
BMI (kg/m^2^)	29.4	29.3	0.88
Ethnicity			0.008
White	61.2	51.2	
Black	14.4	20.1	
Mixed	20	20.9	
Asian	4.4	7.8	
Type of diabetes (1/2)	9.9/90.1	9.4/90.6	0.77
Diabetes duration (years)	17.2	13.2	<0.001
HbA1c (%)	9.23	8.35	<0.001
Smoking (yes)	25.8	22.7	0.25
Hypertension (yes)	80.3	82	0.49
Cardiopathy (yes)	39.5	24.4	<0.001
Dyslipidemia (yes)	71.2	73.5	0.45
Nephropathy (ClCr < 60 ml/min)	29.6	18.8	<0.001
Retinopathy (yes)	69.8	38	<0.001
Visual impairment (yes)	63.3	46.1	<0.001
Foot at risk classification			<0.001
Foot without risk	7.9	48.6	
Neuropathic	52.3	27.2	
Ischemic	4.8	9.9	
Neuroischemic	35	14.3	
Score of symptoms			0.44
Normal/mild	47.3	49.9	
Moderate/severe	52.7	50.1	
Disability score			<0.001
Normal/mild	53.1	80.5	
Moderate/severe	46.9	19.5	
Treatment of neuropathy (yes)	17.3	11.9	0.015
Region			0.001
South/Southeast	65.1	54.6	
Northeast/Midwest	34.9	45.4	

#### Amputation

A significantly higher proportion of men were amputated compared to women (23.5 vs. 6.5 %; p < 0.001). Older patients (60.5 ± 10.8 vs. 57.3 ± 14.6 years; p < 0.001) and those with longer disease duration (17.3 ± 10.4 vs. 13.6 ± 9.5 years; p < 0.001) had also a higher prevalence of amputation, as did patients with type 2 diabetes (14.4 vs. 6.6 %; p = 0.017). Patients of Asian descent were less frequently amputated than those of White, Black or Mixed ethnicities (7.6 % vs. 16.5, 10.8 and 12.2 %, respectively; p = 0.043). Those with retinopathy also had a higher prevalence of amputation (20.5 vs. 7.5 %; p < 0.001), as did patients with visual (17.1 vs. 9.2 %; p < 0.001) or renal impairment (18.4 vs. 9.9 %; p = 0.001). Similar to what was found for ulcer, patients classified as having ischemic foot had a lower prevalence of amputation than those with neuropathic and neuroischemic foot (9.6 vs. 20.6 and 28.7 %, respectively; p < 0.001). Patients with hypertension also had a lower prevalence of amputation (12 vs. 20.4 %; p < 0.001).

Table 3 shows the prevalence of clinical characteristics in patients with and without amputation.

**Table 3:** Prevalence (%) and means of clinical characteristics in patients with and without previous amputation

Variable	Previous amputation		p
	Yes	No	
Age (years)	60.5	57.3	0.001
Sex (female/male)	29.1/70.9	63.3/36.7	<0.001
BMI (kg/m^2^)	28.7	29.4	0.18
Ethnicity			0.043
White	63.3	51.7	
Black	14.8	19.6	
Mixed	18.1	21.1	
Asian	3.8	7.7	
Type of diabetes (1/2)	4.2/95.8	9.5/90.5	0.017
Diabetes duration (years)	17.3	13.6	<0.001
HbA1c (%)	8.76	8.54	0.4
Smoking (yes)	26.1	23.9	0.52
Hypertension (yes)	72.2	83	0.001
Cardiopathy (yes)	36.4	26.8	0.023
Dyslipidemia (yes)	65	74.3	0.022
Nephropathy (ClCr < 60 ml/min)	33.3	19.7	0.001
Retinopathy (yes)	69.8	42.1	<0.001
Visual impairment (yes)	65	47.7	<0.001
Foot at risk classification			<0.001
Foot without risk	3.3	43.9	
Neuropathic	49.5	30.6	
Ischemic	6	9.1	
Neuroischemic	41.2	16.4	
Score of symptoms			0.7
Normal/mild	47.9	49.5	
Moderate/severe	52.1	50.5	
Disability score			<0.001
Normal/mild	39.1	79.2	
Moderate/severe	60.9	20.8	
Treatment of neuropathy (yes)	19.3	11.5	0.005
Region			0.069
South/Southeast	62.4	55.4	
Northeast/Midwest	37.6	44.6	

### Moderate/severe neuropathic symptoms

In the analysis of patients with moderate/severe vs. normal/mild neuropathic symptom scores, those with higher (more severe) scores were more frequently female (53 vs. 45.8 %; p = 0.01), older (58.5 ± 13 vs. 56.4 ± 15.5 years; p = 0.009) and had a higher BMI (29.95 ± 6.58 vs. 28.92 ± 5.79; p = 0.004). White patients were less frequently classified as having severe symptoms than those of Black, Mixed or Asian ethnicities (45.8 vs. 52.9, 54.5 and 57 %, respectively; p = 0.02). Patients with retinopathy had a higher prevalence of severe symptoms (52.7 vs. 45.8 %; p = 0.02), as did those with visual impairment (54.1 vs. 46.1 %; p = 0.006). In contrast to what was found for amputation and ulcer, patients from the emerging regions had a higher prevalence of severe symptoms than those coming from developed regions (57.3 vs. 44.9 %, respectively; p < 0.001).

Table 4 shows the prevalence of clinical characteristics in patients with normal/mild and moderate/severe symptoms.

**Table 4:** Prevalence (%) and means of clinical characteristics in patients with normal/mild and moderate/severe symptoms

Variable	Score of symptoms		p
	Normal/mild	Moderate/severe	
Age (years)	56.4	58.5	0.009
Sex (female/male)	56/63	44/37	0.01
BMI (kg/m^2^)	28.9	29.9	0.004
Ethnicity			0.024
White	58	49.8	
Black	16.9	19.2	
Mixed	18.4	22.4	
Asian	6.7	8.5	
Type of diabetes (1/2)	10.3/89.7	7.9/92.1	0.12
Diabetes duration (years)	13.9	14.4	0.3
HbA1c (%)	8.66	8.46	0.26
Smoking (yes)	25.2	25.4	0.94
Hypertension (yes)	80.1	84.1	0.063
Cardiopathy (yes)	26.4	30.9	
Dyslipidemia (yes)	71.1	75	0.15
Nephropathy (ClCr < 60 ml/min)	22.4	21.5	0.48
Retinopathy (yes)	42.3	49.1	0.024
Visual impairment (yes)	46.1	54.1	0.006
Foot at risk classification			<0.001
Foot without risk	53.8	27.1	
Neuropathic	19.4	41.4	
Ischemic	11.2	5.8	
Neuroischemic	15.5	25.7	
Previous ulceration (yes)	22.3	24.2	0.44
Previous amputation (yes)	12.6	13.3	0.7
Treatment of neuropathy (yes)	11.4	17.9	0.003
Region			<0.001
South/Southeast	64.2	52.1	
Northeast/Midwest	35.8	47.9	

### Independent risk factors for ulcer and amputation

Multivariate regression analyses are summarized in Table 5. Independent risk factors for ulcer were male gender, smoking, foot at risk classification (highest risk associated with the neuroischemic group and lowest to the ischemic group), region of origin (higher risk for those from more economically developed regions), presence of retinopathy and absent vibratory sensation. Risk factors for amputation were male gender, type 2 diabetes, foot at risk classification (higher risk for ischemic foot), hypertension (lower risk), region of origin (South/Southeast), previous history of ulcer and altered vibratory sensation. There was no association between the two outcomes and ethnicity in multivariate analyses.

**Table 5:** Multivariate regression analyses of factors influencing the risk of ulcer and amputation

Factor	OR	95 % CI	p
Ulcer			
Male sex	1.71	1.12; 2.59	0.011
Smoking	1.78	1.09; 2.89	0.019
Neuropathic foot	9.96	4.85; 20.43	<0.001
Ischemic foot	5.64	1.9; 16.7	0.002
Neuroischemic foot	20.34	9.31; 44.38	<0.001
Decreased vibratory sensation	1.36	0.82; 2.24	0.23
Absent vibratory sensation	7.95	4.65; 13.59	<0.001
Retinopathy	1.68	1.08; 2.62	0.022
Region (South/Southeast)	2.39	1.47; 3.87	<0.001
Amputation			
Male sex	2.12	1.2; 3.73	0.009
Type 2 diabetes	3.33	1.01; 11.1	0.049
Neuropathic foot	5.8	1.25; 26.9	0.025
Ischemic foot	19.63	3.43; 112.5	0.001
Neuroischemic foot	11.6	2.43; 55.33	0.002
Decreased vibratory sensation	2.4	1.09; 5.3	0.03
Absent vibratory sensation	3.46	1.64; 7.33	0.001
Hypertension	0.3	0.14; 0.63	0.002
Region (South/Southeast)	2.2	1.1; 4.42	0.027
Previous ulcer (yes)	9.66	4.67; 19.98	<0.001

This is the first large, comprehensive multicenter epidemiological study of foot at risk in diabetes outside Europe [9], Australia [11] or China [12]. We found the prevalence of previous ulcer to be 25 and 14 % for amputation. Additionally, 18 % of patients had an active ulcer at the time of evaluation. Although at a first glance the rate of active foot ulcers could be considered high when compared to the results of other studies (e.g. the FREEMANTLE Study) [11], there are some important differences. Our results are, at this time, from a transversal study and not prospectively evaluated. Additionally, the inclusion of centers with structured foot care units could have contributed to increase active ulcer prevalence. In the next years, in a prospective basis it will be possible to determine with more certainty if the prevalence of ulcers in patients with diabetes in Brazil is higher than in other parts of the world.

Fifty percent had moderate/severe neuropathic symptoms. These findings are in line with previous data [13, 14], especially that from developing nations [15, 16], except for the high proportion of patients with severe symptoms. Also noteworthy, despite the high number of patients with symptoms, we found that only 14 % were receiving medication for neuropathic pain relief.

Our patients had a unique combination of characteristics. The major difference between our patients and those evaluated in studies from the USA [17] or Western Europe [18] is a higher proportion of patients with neuropathic disease, with clear predominance of the neuroischemic population, and a smaller number of patients with isolated ischemic disease. Most studies conducted in economically developed nations show a high prevalence of ischemic disease [19, 20]. Similar findings had been found in one of the centers involved in our study, but with a higher proportion of isolated neuropathic disease [21]. Mean age (57 years old) was also younger than that reported for patients from Western Europe [22] and North America [23]. On the other hand, characteristics like longer disease duration and high frequency of associated comorbidities are in line with data from developed nations [18–23].

Individuals with more severe symptoms were more commonly overweight, older and female. The relation between BMI and symptoms of neuropathy could be associated with other obesity related comorbidities or with excessive load on the lower extremities. Ulcer and amputation were less common in patients of Asian descent, a fact already reported in other studies [24]. However, in multiple adjusted regressions, ethnicity was not a factor associated with either outcome. We believe that this is due to the historic and strong miscegenation typical of our country’s population. Social and economic factors are probably more associated with these ethnic differences in terms of the prevalence of ulcer and amputation [25].

Indeed, ulcer and amputation were more common in the more developed regions of the country, which may be related to the greater prevalence of other diseases such as obesity and metabolic syndrome [26]. Also, social and economic differences might play an important role, in conjunction with disparities in health care access. Increased awareness by health care professionals and more swiftly available access to specialty centers could result in increased diagnosis, which in turn is reflected in an increase in apparent prevalence. Additionally, diabetes prevalence is higher in the South and Southeast regions of the country [27].

Regarding factors independently associated with ulcer risk, the finding that men have an increased risk of ulcer is in accord with the great majority of previous studies [9, 11, 12, 21, 22, 25]. This fact is associated with disease progression and also with social facts related to men being more frequently family providers and consequently showing lower adherence to medical visits and wound care [28].

Loss of vibratory sensation and large fiber damage are frequently seen as the first sensations affected by distal diabetic polineuropathy [29]. Retinopathy is also a long-term complication of diabetes and is expected to co-exist with neuropathy. In addition, it can make self-care and self-examination more difficult and thus predispose to ulcer. In adjusted analysis, however, visual impairment was not an associated risk factor, indicating that microvascular processes occurring simultaneously is a more likely explanation for ulcer than visual deficits.

The lowest risk for ulcer relative to foot at risk classification when compared to foot without risk was isolated arterial disease. The greatest risk was linked to neuroischemic disease. The combination of neuropathy and peripheral arterial disease has been associated with a high ulcer risk in most previous studies [9, 11, 21], but ischemia is normally associated with a greater risk than neuropathy [9, 30]. This difference might be due to neuropathy being more common in developing countries. Similar findings have been published in smaller studies from other emerging nations [12, 15, 16, 21]. A younger age at presentation and a lower prevalence of obesity and other associated diseases apart from diabetes itself may combine to expose patients to ulcer sooner than atherosclerotic disease manifests.

As reported for ulcer, male sex is associated with a greater risk of amputation in almost all studies on diabetic foot complications. In the case of type 2 diabetes, it is more commonly associated with other comorbidities that can predispose to poorer outcomes in ulcer, such as obesity, insulin resistance, dyslipidemia and atherosclerosis [31], thus explaining the greater risk of amputation, even in a younger population. Loss of vibratory sensation and previous ulcer are established risk factors for amputation. Amputation is, in fact, a last resort treatment for diabetic ulcers.

Unlike ulcer, patients presenting ischemic disease, either in an isolated form or associated with neuropathy presented the greatest risk for amputation in multivariate analysis. Ischemic disease has frequently been associated with poorer ulcer outcomes [9, 11], especially when associated with infection [32], explaining the higher probability of ending in amputations. The finding of hypertension being associated with a lower risk for amputation even in adjusted analysis was a surprising one, and no clear explanation could be found for this fact with the current data. Follow up results may cast some light on this matter.

This study has some limitations. The data represent baseline information, and the causative factors here identified have the constrictions inherent to cross sectional evaluations. However, all the risk factors found for ulcer and amputation are well recognized in the literature, which makes us more confident that our estimates are adequate. Another limitation is that we found a relatively high number of patients referred for evaluation who in the end were revealed to present foot without risk. Although serving as a reference category to estimate the risk of neuropathy vs. ischemia for ulcer and amputation, the relatively small number of patients with foot at risk could have underpowered our analysis. Nevertheless, we were able to identify several risk factors associated with those conditions, a detail that reassures us that the analysis deserves merit. Additionally, our population was a large one, and the fact that patients came from several different centers and were primary care referrals support the representativeness of our sample. As with any other studies using ABI for ischemic disease diagnosis, the prevalence of ischemic disease could have been underrepresented, but this methodology has been used in most studies; therefore, data from this study is comparable to that in others. Whether our findings can be extrapolated to other South or Latin American populations is also debatable as our country bears significant ethnic and social differences from others in this region of the world.

## Conclusions

In conclusion, we found a high prevalence of ulcer and amputation in patients with foot at risk in Brazil. Additionally, we found a younger age at presentation and that patients with ulcer presented a higher prevalence of neuropathy compared to ischemic foot at risk. Ischemic disease remains a factor associated with amputations, probably as a determinant of poorer ulcer outcomes, as reported in other studies. Ethnic differences were not of great importance in a miscegenated population, and probably only reflect social and economic differences as well as health care access disparities. We hope the data presented here can contribute to the fast growing literature on diabetic foot and to understanding regional differences in factors affecting the prevalence of ulcer and amputation. This study will continue to follow up patients presenting with ulcer and data on their outcomes are expected in the next couple of years.
